# Short-Term Bone Healing Response to Mechanical Stimulation—A Case Series Conducted on Sheep

**DOI:** 10.3390/biomedicines9080988

**Published:** 2021-08-10

**Authors:** Jan Barcik, Manuela Ernst, Marc Balligand, Constantin Edmond Dlaska, Ludmil Drenchev, Stephan Zeiter, Devakara R. Epari, Markus Windolf

**Affiliations:** 1 AO Research Institute Davos, Clavadelerstrasse 8, 7270 Davos, Switzerland; manuela.ernst@aofoundation.org (M.E.); stephan.zeiter@aofoundation.org (S.Z.); markus.windolf@aofoundation.org (M.W.); 2 Bulgarian Academy of Sciences, Institute of Metal Science ‘Acad. A. Balevski’, Shipchenski Prohod 67, 1574 Sofia, Bulgaria; ljudmil.d@ims.bas.bg; 3 Department of Clinical Sciences, Faculty of Veterinary Medicine, University of Liège, Quartier Vallée 2, Avenue de Cureghem 7A–7D, 4000 Liège, Belgium; marc.balligand@uliege.be; 4 Orthopaedic Research Institute of Queensland, 7 Turner Street, Townsville, QLD 4812, Australia; constantin.dlaska@gmx.at; 5 Institute of Health and Biomedical Innovation, Queensland University of Technology, George Street 2, Brisbane, QLD 4000, Australia; d.epari@qut.edu.au

**Keywords:** bone mechanobiology, fracture healing, interfragmentary motions, active fixation, tissue stiffness

## Abstract

It is well known that mechanical stimulation promotes indirect fracture healing by triggering callus formation. We investigated the short-term response of healing tissue to mechanical stimulation to compare the changes in tissue stiffness during stimulation and resting phases in a preclinical case-series. Four sheep underwent a tibial osteotomy and were instrumented with a custom-made active fixator which applied a mechanical stimulation protocol of 1000 cycles/day, equally distributed over 12 h, followed by 12 h of rest. During each cycle, a surrogate metric for tissue stiffness was measured, enabling a continuous real-time monitoring of the healing progression. A daily stiffness increase during stimulation and an increase during resting were evaluated for each animal. One animal had to be excluded from the evaluation due to technical reasons. For all included animals, the stiffness began to increase within the second week post-op. A characteristic pattern was observed during daily measurements: the stiffness dropped considerably within the first stimulation cycles followed by a steady rise throughout the rest of the stimulation phase. However, for all included animals, the average daily stiffness increase within the first three weeks post operation was larger during resting than during stimulation (Sheep I: 16.9% vs. −5.7%; Sheep II: 14.7% vs. −1.8%; Sheep III: 8.9% vs. 1.6%). A continuous measurement of tissue stiffness together with a controlled fracture stimulation enabled the investigation of the short-term effects of specific stimulatory parameters, such as resting periods. Resting was identified as a potentially determining factor for bone healing progression. Optimizing the ratio between stimulation and resting may contribute to more robust fracture healing in the future.

## 1. Introduction

Most bone fractures consolidate via secondary bone healing by the formation of a fracture callus [1]. It has been long established that the formation of a fracture callus is promoted by mechanical stimulation, which is an induced micromotion in the fracture gap [2]. In clinics, mechanical stimulation is realized by the functional loading of the fractured bone [3]. Experimental animal studies have shown that the formation of a fracture callus is impacted by the amplitude of mechanical stimulation [4,5] and by the size of the fracture gap [5,6]. The relation of the amplitude of stimulation to the gap size is referred to as interfragmentary strain (IFS) [7]. It was reported that in small fracture gaps (2–3 mm), increasing IFS leads to a larger callus response [4,8].

Whereas the impact of IFS on fracture healing is uncontested, it is not entirely understood how the temporal distribution of mechanical stimulation impacts fracture healing [9], although this parameter has direct relevance to postoperative rehabilitation protocols and weight-bearing recommendations. Windolf et al. [10] and Tufekci et al. [11] reported that high fracture activity in the first postoperative weeks accelerates fracture healing in ovine fracture models. In contrast, Claes et al. [12] did not demonstrate the benefit of early dynamization in a rat fracture model and, in another study, demonstrated the advantage of late dynamization [13].

Few studies have aimed to determine the daily optimal temporal distribution of mechanical stimulation. Hente and Perren found that the formation of a fracture callus is modulated by the number of stimulation cycles applied over a day [14,15] and demonstrated in an ovine model that callus size increased with the number of stimulation cycles applied per day [14]. However, their experiments also showed that too frequent stimulation (10,000 cycles per day) inhibited callus formation in the sheep and that sufficient resting time should be provided between stimulation cycles to allow for callus formation [15]. These findings led to the hypothesis that mechanical stimulation works as repeated trauma of the bone and soft tissue [16].

In contrast, Gao et al. [17] tested in a rat model the effect of different resting periods after the application of mechanical stimulation that was induced by low-magnitude high-frequency vibrations. No significant difference was observed in the callus failure load between the animals that received one stimulation batch per day and the animals that received stimulation distributed over three batches per day. Neither did Gardner et al. [18] observe significant differences in callus strength and stiffness when testing different distributions of stimulation (induced by an external actuator) and resting times in a mouse model. It is important to mention that, in both studies, short-term stiffness progression and animal activity were not monitored. Undesired mechanical stimulation that originated from the animal’s activity might have blurred the effects of the controlled stimulation applied in these experiments.

Capturing the isolated effect of mechanical stimulation on fracture healing is challenging in animal experiments due to the parasitic motion in the fracture resulting from the animal’s activity. To circumvent this problem, Tufekci et al. [11] applied controlled interfragmentary motion in a sheep tibia model with an active motorized fixator on a double osteotomy, thus, eliminating the effect of functional loading. In our previous work [19], we further enhanced Tufekci’s fixator by integrating an implanted force sensor to continuously measure the stiffness progression of the repair tissue and developed a control unit that permits the programming of arbitrary stimulation protocols, which are executed autonomously by the active fixator.

In this study, we employed the previously described model [11,19] to apply a well-controlled stimulation protocol composed of stimulation and resting periods in a sheep tibial fracture model, and continuously measured fracture stiffness to investigate the evolution of the mechanical competence of the fracture repair tissue. We aimed to observe the short-term response of the repair tissue with special reference to the role of resting periods in fracture healing. Describing the tissue response to stimulation and to resting will help to better understand the role of the temporal distribution of mechanical stimulation in fracture healing and may further contribute to the optimization of rehabilitation protocols for fracture patients.

## 2. Materials and Methods

### 2.1. Experimental Setup

**Figure 1:**
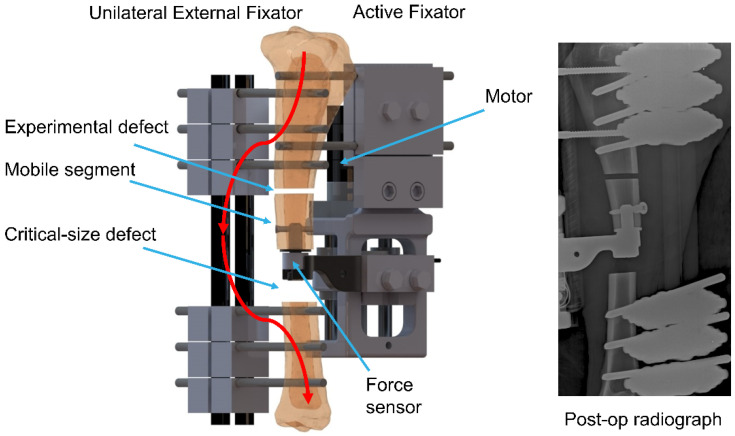
Configuration of the experimental setup. The distal and proximal parts of the tibia are fixed with an external fixator which transfers physiological loading (red line). The mobile fragment is connected to the proximal part via an active fixator that moves it to stimulate and monitor healing in the experimental defect.

The fracture model consisted of a 3 mm experimental defect and a 30 mm critical-size defect in a sheep tibia. These defects were separated by a 30 mm mobile bone segment (Figure 1). The proximal and distal parts of the tibia were fixed with a unilateral external fixator using three 5 mm Schanz screws on either side. The mobile bone segment was fixed to the proximal bone fragment with an active fixator. The unilateral fixator was positioned on the medial aspect of the tibia and the active fixator was fixed to the cranial aspect at 90° to the unilateral external fixator. The active fixator was actuated by a Brushless Direct Current (BLDC) Motor (EC32, Maxon Motor AG, Sachseln, Obwalden, Switzerland) connected to a spindle drive that moved the mobile segment along the bone axis to compress the tissue in the experimental defect in a controlled way. The displacement of the active fixator was measured by an encoder embedded in the motor shaft. The physiological loading that originated from animal weight-bearing was transferred solely via the external fixator (Figure 1) to prevent interference with the mechanical stimulation applied by the active fixator. An implanted force sensor was fixed to the distal aspect of the mobile bone fragment to measure the resistance of the tissue in the experimental defect against compression.

The motion of the active fixator was regulated by a custom-made control unit [19] that was directly attached to the fixator frame and which autonomously executed the programmed stimulation protocol. For each stimulation cycle, the displacement of the mobile bone fragment and the corresponding force was measured and the data was recorded in the internal memory of the control unit.

### 2.2. Animal Experiment

Four female skeletally mature Swiss White Alpine Sheep (age: 5 ± 1.6 years; bodyweight: 75 ± 6 kg) were acclimatized for at least two weeks. Before surgery, a clinical examination, blood analysis and radiographs of the operated tibia ensured good clinical health without pre-existing morbidities. The study was approved by the local ethics committee (Canton of Grisons, Switzerland, approval: TVB 2017_23, 01/09/2017 and conducted under our AAALAC accredited roof. The surgeries were performed on the right tibiae according to the procedure described by Barcik et al. [19], under general anesthesia (sedation with Detomidine, induction with Ketamine and Midazolam and inhalation anesthesia with Sevoflurane) and appropriate analgesia (epidural analgesia with Buprenorphine and with Lidocaine followed by Carprofen and Buprenorphine with Fentanyl patch postoperatively). To minimize the risk of pin-site infection, regular pin cleaning was performed together with antibiotics treatment (Ceftiofur intra- as well as postoperatively for four weeks). The animals were supported in a sling system for the whole period of the study to prevent overloading the osteotomies.

**Figure 2:**
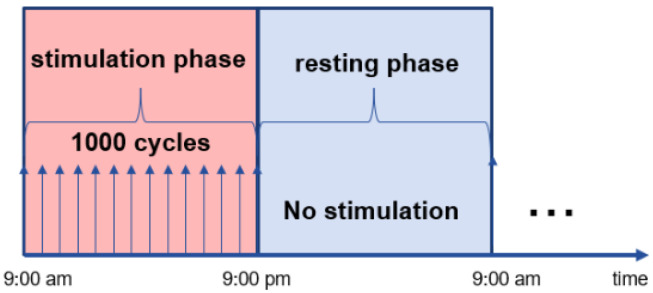
Pictorial representation of the stimulation protocol. Every day, 1000 stimulation cycles were equally distributed from 9:00 a.m. to 9:00 p.m. (stimulation phase) and no stimulation was applied overnight (resting phase). The stimulation protocol was applied from the first day post-op for the entire duration of the study. The arrows represent the applied stimulation and the dots (…) indicate that the same protocol was recuring every day.

Mechanical stimulation was applied by the active fixator from the first day post-op. Each sheep received 1000 stimulation cycles daily with a commanded amplitude of 1.00 mm corresponding to 33% interfragmentary strain. The cycles were equally distributed from 9:00 a.m. to 9:00 p.m. (stimulation phase); no stimulation was applied overnight from 9:00 p.m. to 9:00 a.m. (resting phase) (Figure 2).

The animals were euthanized nine weeks post-op by an overdose of Pentobarbital. The operated bones were dissected, fixed in 70% ethanol, dehydrated and embedded in resin. Subsequently, the specimens were sliced into 100 µm sections and stained with Giemsa-Eosin. A certified veterinary pathologist performed a descriptive qualitative analysis of the stained sections to assess the healing progression and detect potential infection or bone necrosis.

### 2.3. Data Processing and Statistical Analysis

The displacement signal was sampled with 100 Hz and the force sensor signal with 1000 Hz. The force sensor signal was filtered with a recursive application of a Hampel outlier removal (50 repetitions; window size: 5) and smoothed with a moving average filter applied recursively twice (window size: 65). The stiffness of the fracture repair tissue was calculated for each cycle as a quotient of the amplitudes of the force sensor signal and the displacement sensor signal. The stiffness was later filtered on a daily basis with the Savitzky–Golay filter (order: 5, window size: 141). The progression of tissue stiffness was reported in mV/mm (output signal from the force sensor per displacement of the actuator).

Prior to statistical evaluation, the experimental data collected during each day were normalized to the last measurement of the previous day. Based on the data derived, the following parameters were evaluated for the first three weeks post-op:

- Daily minimum stiffness and the stiffness at the end of the stimulation phase—both calculated as the percentage of stiffness at the beginning of the daily stimulation phase (reported as average and standard deviation);
- The daily change in stiffness during the resting ($\Delta_{Resting}$) and stimulation ($\Delta_{stimulation}$) phases, according to Equations (1) and (2) (evaluated for each day, reported as average and standard deviation).

(1) $$\Delta_{Resting}=\frac{\left(x_{f}-x_{l-1}\right)}{x_{l-1}}100\%$$

(2) $$\Delta_{stimulation}=\frac{\left(x_{l}-x_{f}\right)}{x_{f}}100\%$$

where $x_{f}$ is the first data point of the day (the stiffness that was measured at the end of the resting phase—9:00 a.m.), $x_{l-1}$ is the last data point of the previous day (the stiffness that was measured at the beginning of the resting phase—9:00 p.m. on the previous day), and $x_{l}$ the last data point of the day (the stiffness that was measured at the end of the stimulation phase—9:00 p.m.).

The normal distribution of the daily change in stiffness during the resting and stimulation phase was checked with the Shapiro–Wilk test. Using the pared T-Test, we compared the daily changes in stiffness during the resting ($\Delta_{Resting}$) and stimulation ($\Delta_{stimulation}$) phases for each animal for the first three weeks post-op. For all tests, the level of significance was set to α = 0.05. Statistical analysis was performed using SPSS (IBM SPSS^®^ Statistics, Version 23, IBM, Armonk, NY, USA).

## 3. Results

All animals recovered without complication from the surgery and tolerated the fixators and the applied stimulation well. One animal had to be excluded from the evaluation due to a malfunction of the force sensor. In one animal, the data acquisition system failed for a week (Sheep II) and in one animal (Sheep III) the force sensor exhibited a malfunction after Day 24; therefore, subsequent data were excluded.

**Figure 3:**
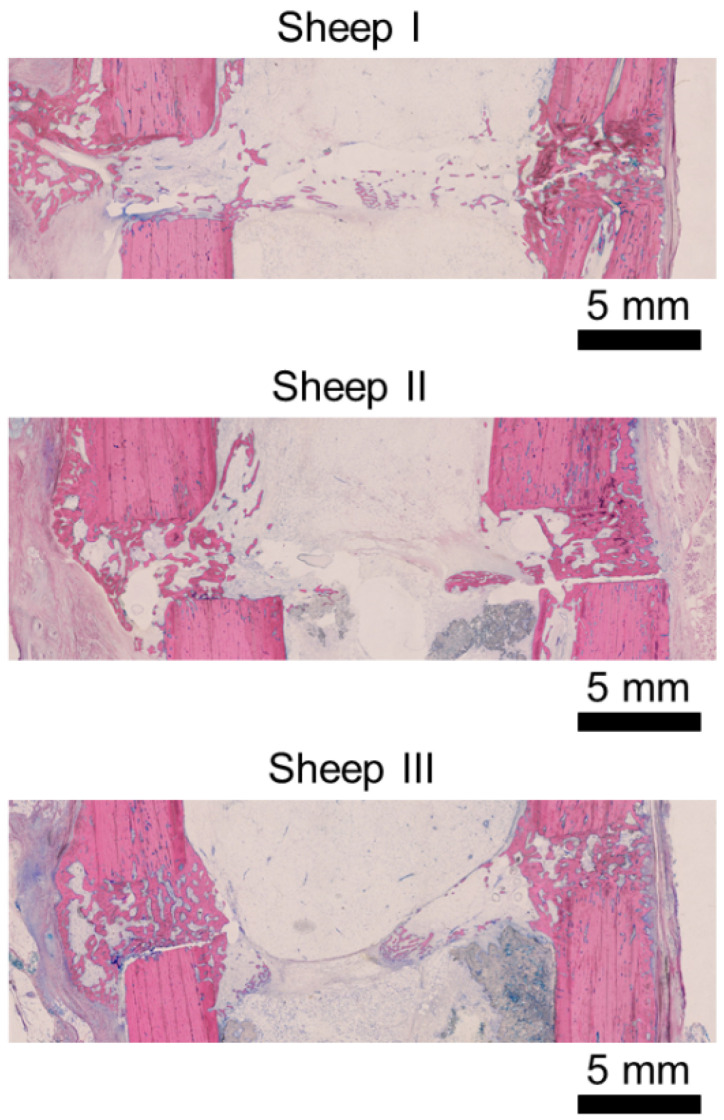
Histological slices displaying vital tissue in the osteotomy. No signs of avascularity nor osteonecrosis were observed. Each slice was provided with a 5 mm scale bar.

The histological analysis confirmed vital bone tissue and bridging at the experimental defect for the included animals and did not reveal any signs of bone infection in the fracture gap nor avascular necrosis of the bone (Figure 3).

### 3.1. Animal I

**Figure 4:**
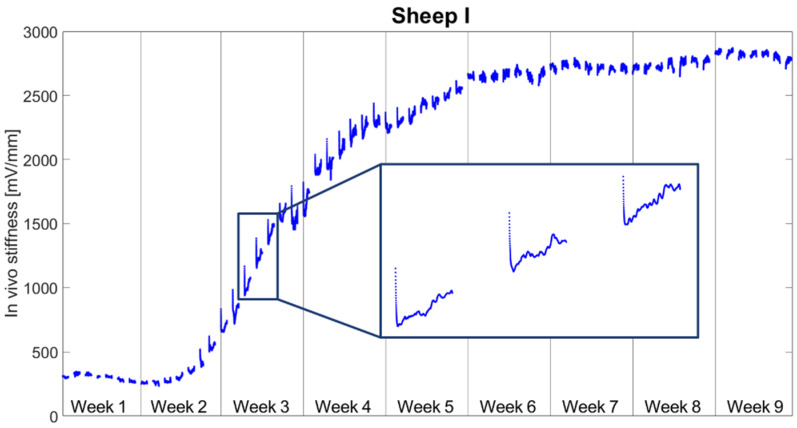
Continuous measurements of the tissue stiffness for Sheep I. Each data point represents one cycle; therefore, each segment shows measurements from 9.00 a.m. to 9.00 p.m.

Figure 4 reports the daily stiffness measurements for Sheep I. Each cluster of points represents data collected during daily cycles from 9:00 a.m. to 9:00 p.m. After an initial plateau phase, the stiffness began to increase at Day 10. From Week 6 onwards, the stiffness plateaued again. In the interval when the stiffness was rising, a characteristic pattern was observed: the stiffness value dropped sharply within the first stimulation cycles of the day followed by a slow rise in stiffness to the end of the 12 h stimulation phase. During the stimulation phase, the stiffness decreased until reaching an average of 87.3 ± 7.9% and rose until the end of the day to the level of 94.3 ± 7.3% of the initial daily stiffness.

**Figure 5:**
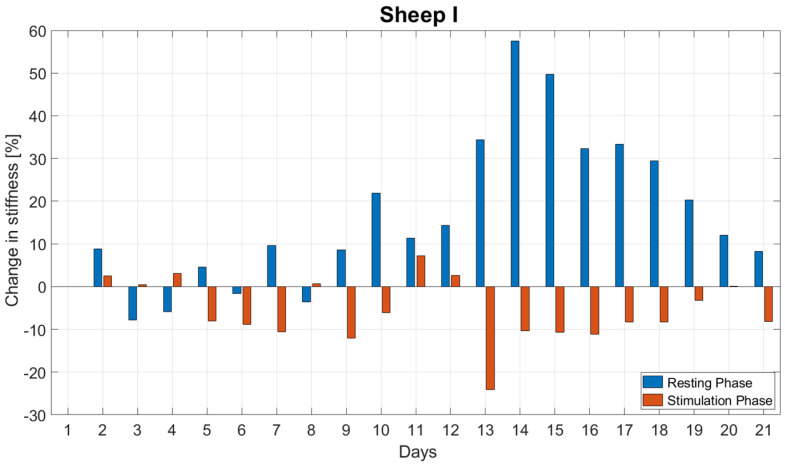
Daily changes in stiffness during the resting and stimulation phases for the first three weeks post-op for Sheep I. The stiffness change during resting was significantly greater than during stimulation (*p* < 0.001).

The stiffness increased during the resting phases on average by 16.9 ± 17.9% per day. In contrast, during stimulation it decreased on average by 5.7 ± 7.3% per day (Figure 5). The stiffness change during resting was significantly greater than during stimulation (*p* < 0.001).

### 3.2. Animal II

**Figure 6:**
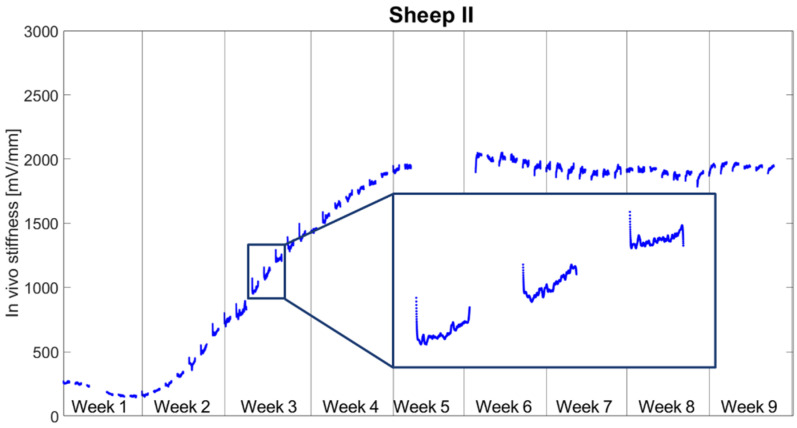
Continuous measurements of the tissue stiffness for Sheep II. Each data point represents one cycle; therefore, each segment shows measurements from 9.00 a.m. to 9.00 p.m.

Figure 6 reports the daily stiffness measurements for Sheep II. After a slight drop in tissue stiffness within the first week post-op, the stiffness began to increase at Day 8 post-op and plateaued around Week 5. As with Sheep I, the characteristic daily pattern was observed in the interval when the stiffness was rising—an abrupt drop followed by a slow increase throughout the stimulation. Due to technical issues with the data acquisition system, the data from Week 5 is missing. During the stimulation phase, the stiffness decreased until reaching an average of 89.5 ± 5.1% and rose until the end of the day to the level of 98.3 ± 5.1% of the initial daily stiffness.

**Figure 7:**
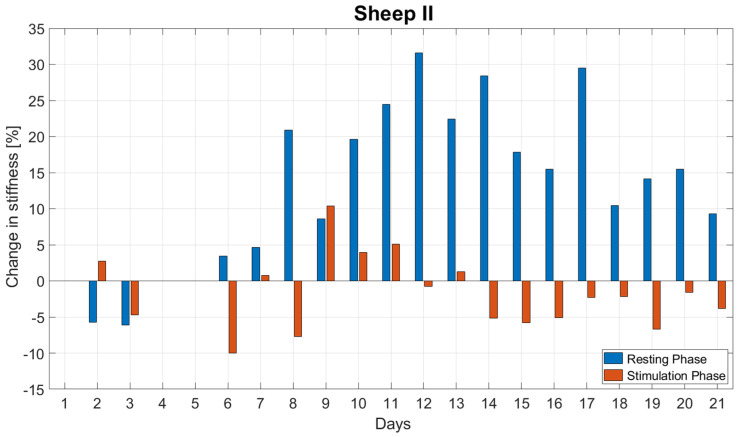
Daily changes in stiffness during the resting and stimulation phases for the first three weeks post-op for Sheep II. The stiffness change during resting was significantly larger than during stimulation (*p* < 0.001).

The normalized stiffness increased during the resting phases on average by 14.7 ± 11.1% per day and decreased during the simulation phases on average by 1.8 ± 5.1% per day (Figure 7). The stiffness change during resting was significantly greater than during stimulation (*p* < 0.001).

### 3.3. Animal III

**Figure 8:**
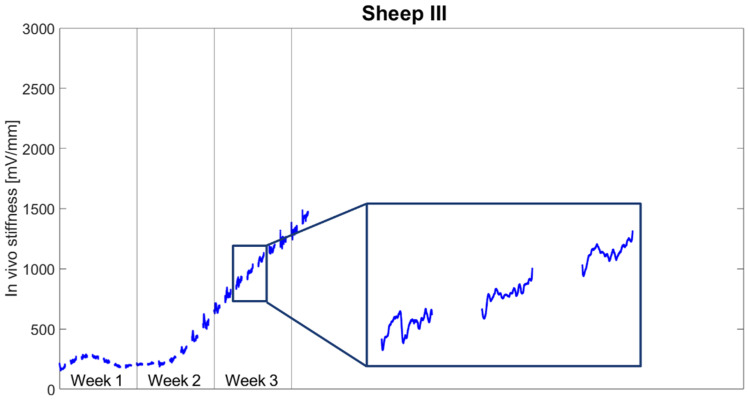
Continuous measurements of the tissue stiffness for Sheep III. Each data point represents one cycle; therefore, each segment shows measurements from 9.00 a.m to 9.00 p.m.

**Figure 9:**
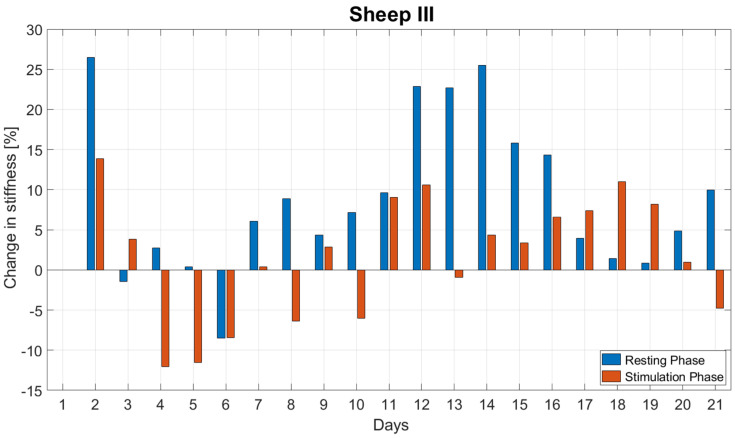
Daily changes in stiffness during the resting and stimulation phases for the first three weeks post-op for Sheep III. The stiffness change during resting was significantly greater than during stimulation (*p* = 0.003).

Figure 8 reports the daily stiffness measurement for Sheep III. Following minor fluctuations during the first week, stiffness started to continuously increase from the 10th day post-op until the force sensor broke 24 days post op. The stiffness drop pattern as observed in both previous sheep was less frequently present in this animal. During the stimulation phase, stiffness decreased until reaching an average of 91.6 ± 4.3% of the initial daily stiffness and rose until the end of the day to 101.6 ± 7.7% of the initial daily stiffness. The normalized stiffness increased on average by 8.9 ± 9.6% per day during the resting phases and on average by 1.6 ± 7.7% per day during the stimulation phases (Figure 9). The stiffness change during resting was significantly greater than during stimulation (*p* = 0.003).

## 4. Discussion

In this in vivo study, we investigated the short-term response of fracture repair tissue to mechanical stimulation by continuously measuring a surrogate metric for tissue stiffness during the healing process to identify sub-daily stiffness variations. Stiffening initiated during the second week after surgery. Fracture stiffness gradually increased until reaching a plateau between Weeks 5 and 6 post-op. However, continuous monitoring revealed that tissue stiffness did not steadily increase through the stimulation and resting periods. A decrease in stiffness was often observed during stimulation periods.

In line with these results, Hente and Perren [15] pointed out that resting time between stimulation cycles might be a crucial and underestimated parameter that determines fracture healing. In their study, continuous intermittent stimulation with short resting periods (<9 s) inhibited callus formation, whereas resting periods of 2.4 h allowed for the robust formation of a fracture callus. This stimulation protocol was different to our study. We applied a stimulation pattern resembling a simplified clinical situation with an active phase during the day (12 h) and resting overnight (12 h). During the active phase, stimulation cycles were applied every 44 s. We observed that, after an initial drop, the daily stiffness was still increasing until the end of the stimulation period. Hente and Perren used a different model. A wedge-shaped bone fragment was tilted around its apex; thus, generating a strain gradient along the edges of the fragment. In contrast, our model created an axial movement across the fracture plane. To minimize the effect of functional loading on the experimental osteotomy, Hente and Perren further performed a tenotomy on the Patellar and Achilles tendons. Similarly, Augat et al. [20] and Bishop et al. [21] cut the Achilles tendon. However, these approaches only temporarily prevent functional loading. When the tendons start healing again, the animal may again load the fractured limb. This effect was excluded in our model by applying the active fixator on a double osteotomy, eliminating load transfer through the critical-size defect into the isolated bone segment. However, this model was also not completely free of undesired loading effects. In Sheep III, the critical-size defect was found to be shortened due to the deformation of the external fixator. Some load transfer by contact in the defect between bone and force sensor cannot be excluded, which might have caused the observed failure of the force sensor 24 days post-op.

The observed stiffness difference between the stimulatory phases was due to an apparent stiffness drop during the first stimulation cycles of the day. The drop was predominant in the stiffening phase. This suggests that the drop was not an artefact of the measurement system but appears to be an inherent characteristic of the model’s healing process. In previous studies [22,23], where mechanical stimulation was realized by cyclic loading applied in a bundle once a day, a similar drop was observed within the first 100 cycles. Meyers at al. [23] attributed these changes to the preferential flow of interstitial fluid. Thus, during our overnight resting periods, the repair tissue in the fracture gap might have attracted fluid, which was squeezed out again by the applied stimulation. Such behavior is known from other musculoskeletal tissues as, for example, cartilage or intervertebral disc, which shrink during the day due to constant physiological compression and regain shape during overnight rest [24].

On the other hand, the drop might reflect the destruction of delicate, newly formed structures during the resting period due to the resumption of fracture stimulation. Perren [16] hypothesized that the effect of mechanical stimulation is based on repeated trauma of the bone and soft tissue. Thus, alternating stimulatory and resting periods could function as a trigger to further induce repair processes. Both hypotheses are not mutually exclusive and the observed effect could be a combined result.

After the daily initial drop in stiffness, stiffness rose until the end of the stimulation phase. Hente and Perren [15] demonstrated that the minimum recovery time that would allow for callus formation may lie between 9 and 86 s. Therefore, the slow rise in stiffness in our study might reflect a healing cascade taking place with relatively short recovery intervals of 44 s between loading cycles. Moreover, Hente and Perren reported in the same study that the intensity/frequency of stimulation impacts the formation of fracture callus and the progression of stiffness during the course of healing.

Since stimulation was only executed during day time, it cannot be ruled out that certain metabolic effects related to the natural day–night cycle [25] also contributed to the observed stiffening variations.

The results of this study are reported in the form of a descriptive case series; therefore, no sample size calculation was performed in the context of the presented data. Data from all animals included were statistically evaluated only for the first three weeks post-op, because this time period was seamlessly captured for all included animals, thus, enabling the comparison of results between them.

During previous experiments by our group [19], we observed that the stiffness measurements performed by the fixator may have underestimated the absolute tissue stiffness [19]. This was caused by the relatively high structural compliance of the active fixator due to the long moment arms between the active fixator and the bone. Therefore, we did not report absolute tissue stiffness but, instead, we circumvented the impact of compliance on the experimental data by normalizing the data collected during each day to the last measurement of the previous day.

Our study is the first in which the fracture healing process was monitored with such a high temporal resolution. This approach enabled the quantification of the short-term (sub-daily) response of fracture-healing tissue to mechanical stimulation and the comparison of the increase in tissue stiffness during stimulation and during resting. These novel data underline the importance of the resting phases within mechanical stimulation as a crucial parameter for robust stiffening of the healing tissue.

Besides its scientific value for advancing our knowledge of bone healing processes, the reported findings might have basic clinical implications for designing future fracture rehabilitation protocols. The approach has the potential to optimize the temporal distribution of mechanical stimulation, as outlined in [26] and offers future opportunities to determine the optimum ratio and arrangement of stimulation and resting periods to support timely and robust healing.

## 5. Conclusions

In this manuscript, we reported the continuous monitoring of fracture repair tissue by means of a surrogate metric for tissue stiffness. The experimental data revealed that the stiffness of the repair tissue increased predominantly during resting periods. While the role of mechanical stimulation in indirect fracture healing was uncontested, these results highlight the equally important role of resting periods in the bone healing cascade. To achieve robust healing, stimulation should be well-orchestrated with resting. However, further experiments are required to determine the most optimal ratio of stimulation vs. resting.

## Data Availability

Not applicable.
